# Anthocyanin-Rich Juice Lowers Serum Cholesterol, Leptin, and Resistin and Improves Plasma Fatty Acid Composition in Fischer Rats

**DOI:** 10.1371/journal.pone.0066690

**Published:** 2013-06-18

**Authors:** Daniela Graf, Stephanie Seifert, Anke Jaudszus, Achim Bub, Bernhard Watzl

**Affiliations:** Department of Physiology and Biochemistry of Nutrition, Max Rubner-Institut, Federal Research Institute of Nutrition and Food, Karlsruhe, Germany; Sapienza University of Rome, Italy

## Abstract

Obesity and obesity-associated diseases e.g. cardiovascular diseases and type 2 diabetes are spread worldwide. Anthocyanins are supposed to have health-promoting properties, although convincing evidence is lacking. The aim of the present study was to investigate the effect of anthocyanins on several risk factors for obesity-associated diseases. Therefore, Fischer rats were fed anthocyanin-rich grape-bilberry juice or an anthocyanin-depleted control juice for 10 weeks. Intervention with anthocyanin-rich grape-bilberry juice reduced serum cholesterol and tended to decrease serum triglycerides. No effects were seen for serum non-esterified fatty acids, glucose, and insulin. Anthocyanin-rich grape-bilberry juice intervention reduced serum leptin and resistin, but showed no influence on serum adiponectin and secretion of adipokines from mesenteric adipose tissue. Furthermore, anthocyanin-rich grape-bilberry juice increased the proportion of polyunsaturated fatty acids and decreased the amount of saturated fatty acids in plasma. These results indicate that anthocyanins possess a preventive potential for obesity-associated diseases.

## Introduction

In Western countries with high prevalence of obesity and its associated diseases, cardiovascular diseases and diabetes are severe public health problems. As obesity is spreading worldwide, it is expected that the incidence of these diseases will increase over the coming years [1].

Anthocyanins, a subgroup of flavonoids, occur in red, violet, and blue fruits and vegetables, especially berries. Several health-promoting properties are discussed for anthocyanins, including anti-diabetic properties and beneficial effects on the cardiovascular system [2]. Two rodent and one human study could show glucose and/or insulin-lowering effects of anthocyanins [3]–[5]. Data on the effects of anthocyanins on cholesterol and triglyceride levels, which are risk factors for cardiovascular diseases, are rather inconsistent. In several human and animal studies cholesterol and/or triglyceride-lowering effects occurred, while other studies showed no influence [6]–[9] and in one animal study the anthocyanin intervention even increased serum triglycerides and cholesterol [10].

The development of cardiovascular diseases, particularly atherosclerosis, is associated with endothelial dysfunction. Saturated fatty acids (SFA) are known to impair, whereas long-chain n3-polyunsaturated fatty acids (PUFA) improve endothelial function [11]. So far only one intervention study investigated the influence of anthocyanins on plasma fatty acid composition using a background diet with low fat content (10%), but high in PUFA. Supplementing this diet with anthocyanins increased the amount of the long-chain n-3-PUFA EPA and DHA in plasma of rats [12]. Furthermore, it has been reported, that EPA and DHA concentrations are increased in humans with regular wine consumption, regardless of the wine color [13].

The adipose tissue secretes several mediators, called adipokines (e.g. adiponectin, leptin, and resistin), which have regulatory functions in the metabolism. Additionally, it has been reported that adipokines are involved in the development of metabolic diseases, such as type 2 diabetes and cardiovascular diseases. High levels of leptin and resistin, occurring in obese individuals, promote the development of insulin resistance, whereas adiponectin seems to prevent insulin resistance [14]. For a detailed description of the effects of adipokines the reader is referred to the review of Ouchi *et al.* [14]. The human visceral adipose tissue seems to be important in the development of obesity-associated diseases, especially diabetes. Because of the portal drainage, only the mesenteric adipose tissue (MAT) in rodents resembles human visceral adipose tissue [15]. So far, it is unknown how anthocyanins affect the secretory properties of MAT.

The aim of the present study was to investigate the effect of a 10-week intervention with anthocyanins on several risk factors for diabetes and cardiovascular diseases, as well as on plasma fatty acid composition. One focus of the present study was to mimic the situation in Western countries. Therefore, we used a diet which ensured a Western-style fat intake and focussed on MAT as fat depot. Fischer rats were fed a diet containing 34% energy from fat with high content of SFA, which closely resembles fat intake in Western countries, and received an anthocyanin-rich grape-bilberry juice (ARJ).

## Materials and Methods

### Ethics statement

This study was approved by the Animal Care Committee of the Regional Administrative Authority Karlsruhe (Permit Number: 35-9185.81/G-221/10) and all animal care and handling were conducted in strict accordance with the guidelines of the German law on animal care.

### Animals and diets

Male Fischer 344 rats at the age of 10 weeks were purchased from Charles River Laboratories (Sulzfeld, Germany). Rats were housed two animals per cage under temperature- (21±2°C) and humidity-controlled (55–65%) conditions with a 12-h-light/-dark cycle. After one week of adaption animals were randomly assigned to intervention groups (n = 30/group). Blood and organs from six animals per group were used to assess bioavailability of anthocyanins. The anthocyanin group received ARJ, containing 1551 mg anthocyanins/L, the control group received polyphenol-depleted grape-bilberry juice. Pure juices were the only sources of liquids. The major anthocyanins were malvidin-3,5-diglucoside and peonidin-3,5-diglucoside. The composition of the juices and data of anthocyanin analysis has been described in detail elsewhere [16]. Juices were produced by the Geisenheim Research Centre. The juices were provided daily via the drinking bottle, right before the beginning of the 12-h-dark cycle, in order to ensure maximum light protection of anthocyanins. The daily juice consumption was recorded. Rats were fed a diet based on AIN93G containing 17% (wt:wt) lard (Ssniff, Soest, Germany), accounting for 34% energy. Due to its fatty acid composition with high SFA and low PUFA, lard was chosen as fat source. Rats received diet and juice *ad libitum*. Body weight and food intake were recorded four times a week. After 8 weeks of intervention, rats of the bioavailability group were housed individually in metabolism cages for 24 h, to collect urine and feces. After 10 weeks of intervention, rats were feed-deprived overnight, anesthetized by exposure to CO_2_, and killed by decapitation. Blood was collected and processed for serum and plasma. The mesenteries were collected and mesenteric adipose tissue (MAT) was isolated. Livers were frozen immediately on dry ice and stored at −80°C until further analysis.

### Sample preparation

#### Short term culture of MAT specimens

For measuring the adipokine release of MAT the method of Paul *et al.* [17] was used with slight modifications. Briefly, the mesenteric lymph nodes, blood vessels, and soft tissue were removed, and adipose tissue fragments were prepared under sterile conditions. 200–240 mg of adipose tissue fragments were incubated at 37°C and 5% CO_2_ in 2 mL DMEM F12 (Invitrogen, Darmstadt, Germany) supplemented with 100 mL/L heat-inactivated fetal bovine serum (PAA, Cölbe, Germany), 2 mmol/L L-glutamine, 500 U/L penicillin, and 50 µg/L streptomycin (all Invitrogen). After 3 h of incubation, supernatants were collected and stored at −20°C until measurement of adipokines. For each rat, sample preparation was accomplished in triplicate.

#### Preparation of serum

For serum preparation blood was collected and the clotted blood was centrifuged at 2000 x g for 10 min. Aliquots of serum were stored at −20°C until further analysis.

#### Preparation of plasma

For plasma preparation blood was collected in heparinised tubes and centrifuged at 400 x g for 10 min. 0.1 mg BHT/mL plasma was added and aliquots were stored at −20°C until further analysis.

### Quantification of adipokines

For the quantification of adiponectin (AdipoGen, Liestal, Switzerland), leptin, and resistin (BioVendor, Heidelberg, Germany) in tissue culture supernatant or serum, sandwich ELISAs were performed according to the manufacturer's instructions. For quantification of adipokines in MAT supernatant, the triplicates were pooled and analysed in duplicates.

### Measurement of glucose, insulin, cholesterol, non-esterified fatty acids (NEFA), and triglycerides in serum

Glucose (Gluco-Quant, Roche, Mannheim, Germany), insulin (Rat insulin ELISA, Millipore, Schwalbach, Germany), cholesterol (CHOD-PAP, Roche), NEFA (NEFA-HR2, Wako, Neuss, Germany), and triglycerides (GPO-PAP, Roche) were measured using commercial kits according to the manufacturers' instructions.

### Measurement of fatty acid profiles in plasma

For analysis of plasma fatty acids a GC method with flame ionization detection was used, which separated FAME ranging from C4 to C26. The detailed method was described earlier [18].

### Statistical analysis

For statistical analysis of effects between the two groups t-tests were performed. If data failed to satisfy variance homogeneity or normal distribution, the Mann-Whitney Rank Sum test was employed. SigmaPlot 11.0 (Systat Software, Erkrath, Germany) was used for all statistical analyses. Differences were considered statistically significant when p < 0.05. All data are presented as mean ± standard deviation.

## Results

### Body weight and food consumption

Data on food, juice, and anthocyanin intake as well as body and organ weights have been presented previously [16]. In brief, neither food and juice intake, nor body and organ weights differed between the groups. Anthocyanin intake in the ARJ group was 15 mg anthocyanins/day, which corresponds to approximately 50 mg/kg body weight, whereas anthocyanin intake of the control group was less than 0.01 mg/day.

### Glucose, insulin, cholesterol, NEFA, and triglycerides in serum

The intervention with ARJ did not influence serum glucose and insulin levels (**Table 1**). Cholesterol concentrations were reduced after 10 weeks of ARJ consumption (**Table 1**). ARJ also tended to decrease triglyceride concentrations (p = 0.08). Serum NEFA levels were not influenced by the intervention with ARJ (**Table 1**).

**Table 1:** Concentrations of glucose, insulin, cholesterol, NEFA, and triglycerides in serum of Fischer rats after 10 weeks of intervention with polyphenol-depleted grape-bilberry juice (control) or ARJ (n = 24/group).

	Control	ARJ	p-value
Glucose *g/L*	1.4±0.1	1.4±0.2	0.253
Insulin *μg/L*	2.9±1.6	3.6±2.5	0.464
Cholesterol *g/L*	1.1±0.1	1.0±0.1	0.029
NEFA *mmol/L*	0.65±0.21	0.67±0.17	0.614
Triglycerides *g/L*	3.4±1.0	2.9±0.8	0.079

ARJ: Anthocyanin-rich grape-bilberry juice

### Adipokines in serum and MAT culture supernatant

**Figure 1:**
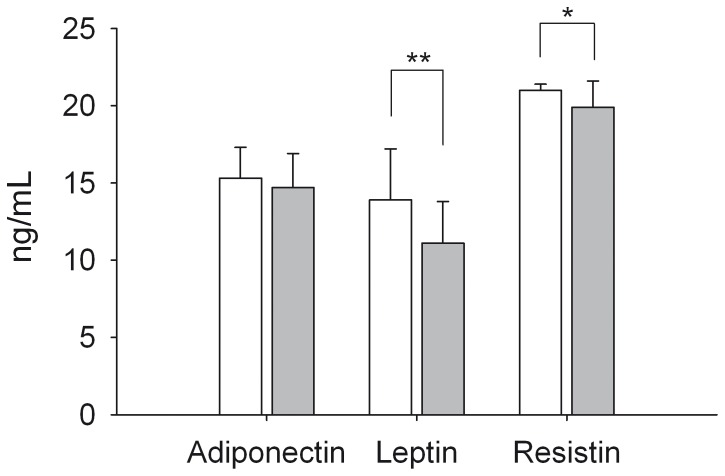
Serum adipokine concentrations. Concentration of adiponectin, leptin, and resistin in serum of Fischer rats after 10 weeks of intervention with control juice (white bars) or ARJ (grey bars; n = 24/group).

The adipokines adiponectin, leptin, and resistin were quantified in serum and MAT culture supernatant. Rats of the ARJ group had lower serum leptin and resistin levels compared to the control group. Adiponectin serum levels did not differ between ARJ and control group (**Figure 1**).

The intervention did not influence the adipokine concentrations in tissue culture supernatants of MAT (**Table 2**).

**Table 2:** Concentrations of the adipokines adiponectin, leptin, and resistin in tissue culture supernatants of MAT of Fischer rats after 10 weeks of intervention with polyphenol-depleted grape-bilberry juice (control) or ARJ (n = 24/group).

	Control	ARJ	p-value
Adiponectin	3.7±0.6	3.9±0.6	0.21
Leptin	26.9±5.0	25.5±5.2	0.36
Resistin	13.2±4.0	13.1±2.8	0.78

ARJ: Anthocyanin-rich grape-bilberry juice

### Fatty acids in plasma

**Figure 2:**
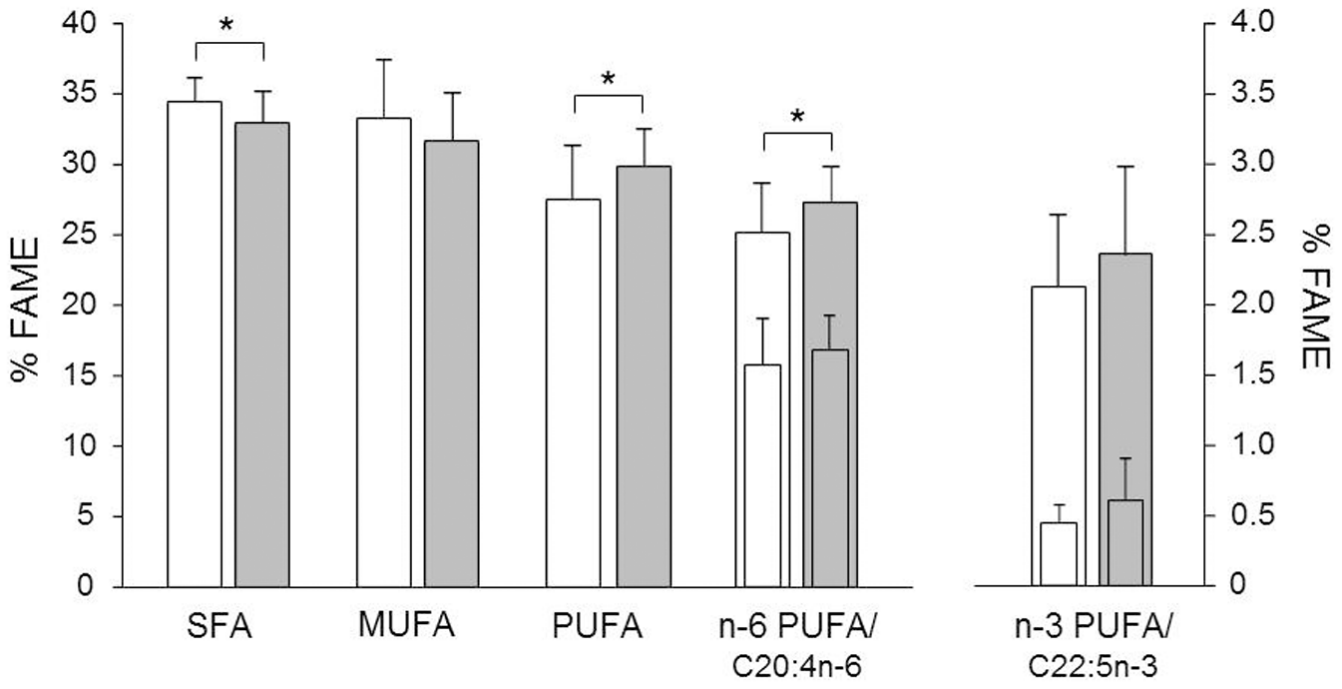
Fatty acid spectra in plasma. Percentage of SFA, MUFA, and PUFA of total plasma fatty acids of Fischer rats after 10 weeks of intervention with polyphenol-depleted grape-bilberry control juice (white bars) or anthocyanin-rich juice (grey bars; n = 24/group). The smaller bars show the fatty acids with the n-6/n-3-PUFA with the largest increases. FAME: fatty acid methyl ester. * p<0.05

The distribution of fatty acids was determined in plasma (**Table 3**). As shown in **Figure 2**, the treatment with ARJ lowered the percentage of SFA and enhanced PUFA in plasma. The reduced SFA are mainly due to a decrease in palmitic acid (C16:0), whereas for PUFA the highest increases were seen for arachidonic (C20:4n-6) and linoleic acid (C18:2n-6). Nonetheless, the n-6/n-3-ratio did not differ between ARJ and control group, since several n-3 PUFA increased, too.

**Table 3:** Major fatty acids^1^ [% FAME] in plasma of Fischer rats after 10 weeks of intervention with polyphenol-depleted grape-bilberry juice (control) or ARJ (n = 24/group).

	Control	ARJ	p-value
C14:0	0.70±0.17	0.51±0.07	<0.001
C16:0	23.74±2.27	21.98±1.63	0.003
C16:1*c*9	3.12±0.88	2.18±0.51	<0.001
C18:0	9.08±2.07	9.54±1.89	0.529
C18:1*c*9	25.98±3.69	25.64±3.02	0.975
C18:1*c*11	3.01±0.34	2.63±0.34	<0.001
C18:2n-6	7.30±1.07	8.19±0.97	0.004
C20:4n-6	15.78±3.28	16.86±2.43	0.201
C22:4n-6	0.57±0.09	0.62±0.10	0.039
C22:5n-6	0.76±0.14	0.83±0.16	0.062
C22:5n-3	0.45±0,13	0.61±0.31	0.101
C22:6n-3	0.95±0.27	1.03±0.22	0.089
n-6/n-3	12/1	12/1	0.990

ARJ: Anthocyanin-rich grape-bilberry juice

1 >0.5% FAME

## Discussion

In the present study, the influence of an ARJ intervention on carbohydrate and lipid metabolism, serum concentrations and MAT secretion of adipokines, and fatty acid profil in plasma was investigated. As we intended to determine the preventive potential of an anthocyanin-rich diet, we decided to use a whole food in form of a juice and not to use supplements for this intervention. Rats of the anthocyanin group ingested approximately 15 mg total anthocyanins per day, which corresponds to 50 mg/kg body weight. As discussed in Graf *et al.* [16], this can rather be seen as a physiological and not pharmacological dose, which led to plasma anthocyanin concentrations in the low nanomolar range. Further bioavailability data are presented elsewhere [16].

For anthocyanins anti-diabetic properties are assumed and human as well as animal studies reported increased insulin sensitivity or lower blood glucose concentrations after an anthocyanin intervention [3]–[5]. In contrast to these findings, serum glucose and insulin concentrations were not influenced by the intervention in the present study. Although rats had free access to juice until the section, which normally results in higher glucose and insulin concentration compared to fasting rats, serum glucose levels were rather low in both groups (1.4 g/L) compared to the reference values for age-matched Fischer rats (0.9–3.6 g/L with an average of 2.2 g/L; given by Charles River). Therefore, it seems consequential that in the present study no direct effects of anthocyanins on glucose and insulin concentrations in serum were observed.

Dyslipidemia, especially elevated cholesterol and triglyceride levels, is a risk factor for cardiovascular diseases [19]. Data on the impact of anthocyanins on cholesterol and triglyceride levels are inconsistent [6]–[10]. Our results indicate that consumption of ARJ can decrease serum concentrations of cholesterol and triglycerides. Cholesterol concentrations are influenced by many factors: intestinal cholesterol absorption, hepatic cholesterol syntheses, biliary excretion, and cellular use [20]. One possible mechanism for cholesterol lowering effects of anthocyanins could be the inhibition of cholesterol synthesis. It has been shown that anthocyanins can activate AMPK [5], [21], which is involved in the regulation of energy homeostasis and influences the activity of many enzymes. One enzyme, which is inhibited by AMPK, is HMG-CoA reductase [22]. As HMG-CoA reductase is the limiting enzyme of cholesterol synthesis, increased AMPK activity would inhibit cholesterol synthesis and consequently lead to lower cholesterol levels. Furthermore, AMPK inhibits the activity of acetyl-coA carboxylase (ACC) 1 and ACC-2, which leads to increased fatty acid oxidation and decreased fatty acid synthesis [22], and, accordingly, lower triglyceride concentrations. Guo *et al*. [21] could show an increased AMPK activity which led to decreased ACC activity and increased fatty acid oxidation *in vitro* after incubating HepG2 cells with cyanidin-3-O-β-glucoside. In light of this, it seems possible that cholesterol and triglyceride lowering effects of anthocyanins *in vivo* are mediated *via* activation of AMPK. Therefore, future studies should investigate effects of anthocyanins on AMPK expression and activity.

We further examined the influence of ARJ consumption on fatty acids in plasma. Although the diet was rich in SFA, SFA decreased and PUFA increased in plasma after 10 weeks of ARJ consumption. It has been described by another study that anthocyanin interventions can increase the plasma concentrations of the long chain n-3-PUFA EPA (C20:5n-3) and DHA (C22:6n-3) [12]. Both studies differed in many details, but both indicate that anthocyanins can increase PUFA concentrations in plasma. Several beneficial effects of both n-3 and n-6 PUFA have been described. As PUFA are incorporated in cell membranes, they influence the membrane fluidity [23], and might therefore have a positive influence on vascular function [24], [25]. Our results might be a cautious indication that anthocyanins mediate cardioprotective properties through their influence on plasma fatty acid composition. Consequently, future studies should also investigate the influence of anthocyanins on functional membrane fatty acids. Additionally, it is postulated that PUFA have anti-inflammatory effects [26]. Our results suggest that anthocyanins might lead to an altered fatty acid profile with reduced inflammatory potential. As low grade chronic inflammation is involved in the pathogenesis of most obesity-associated diseases, these anthocyanin-related effects on plasma fatty acids should be further investigated.

The intervention with ARJ resulted in reduced serum concentrations of leptin and resistin, whereas no effect was observed for adiponectin. Data on the influence of anthocyanins on adipokines are rather controversial. *In vivo* studies with anthocyanins have reported increased, lowered, as well as unchanged adiponectin concentrations [6], [8], [27], [28]. For leptin, both, a lowering effect or no effects have been described [28], [10]. Due to the fact that sources of anthocyanins, background diets, and populations vary in these studies, it is impossible to identify the causes for the different outcomes.

Leptin as well as resistin are elevated in obesity [29]–[31]. It has been reported that resistin is involved in the pathogenesis of obesity-associated insulin resistance [32]–[34]. Consequently, the reduced resistin concentration after ARJ consumption might contribute to a lower risk for the development of diabetes.

In contrast to serum concentrations of adipokines, no effect was observed for the secretory activity of MAT regarding adiponectin, leptin, and resistin. Guo *et al.* [27] reported increased gene expression of adiponectin in epididymal adipose tissue of mice after intervention with cyanidin-3-glucoside. So far, no study analysed the secretory activity or gene expression in MAT. In contrast to other adipose tissues, MAT drains into the portal vein [35]. Therefore, rodent MAT can be considered as a suitable model for human visceral adipose tissue, which seems to be involved in the development of obesity-associated diseases in humans [36], [37]. As different adipose tissues vary in their endocrine functions, further studies should investigate the secretory activity of various adipose tissues.

Our results indicate a positive influence of anthocyanins on serum adipokines, which seems to be independent of MAT, the central visceral adipose tissue. Further studies are needed to determine which adipose depots are responsible for the observed changes in serum and under which conditions anthocyanins exert this effect.

In conclusion, a 10 week intervention with ARJ and a diet, which mimicked Western fat intake led to changes in plasma fatty acids as well as to reductions in serum cholesterol, leptin, and resistin concentrations. These results indicate that intake of anthocyanins at physiological doses e.g. via complex foods like juices, might have a preventive potential for obesity-associated diseases such as cardiovascular disease or type 2 diabetes.
